# CO2 production in waste management during the COVID pandemic in an Italian hospital: Mattia Di Russo

**DOI:** 10.1093/eurpub/ckac130.071

**Published:** 2022-10-25

**Authors:** M Di Russo, A Heidar Alizadeh, G Santoli, C Castagna, M Nurchis, A Rosano, C Cadeddu

**Affiliations:** 1 Department of Life Sciences and Public Health, Catholic University of Sacred Heart, Rome, Italy; 2 Italian Institute for Planetary Health, Rome, Italy; 3 National Institute for Public Policy Analysis, Rome, Italy; 4 Department of Woman and Child Health and Public Health, Catholic University of Sacred Heart, Rome, Italy

## Abstract

**Background:**

Personal protection equipment (PPE) use in hospitals has consistently increased due to the Sars-Cov-2 outbreak, in wards repurposed for Covid-19 patients and wards that kept their usual activity. This increase influenced an environmental emergency in terms of health waste (HW) disposal. This study aims to assess the economic and environmental impact of the increase in HW generated before and during the pandemic in an Italian Hospital.

**Methods:**

Data from 2016 to 2019 and 2020 to 2021 was retrieved from Risk Management department. Per capita and per days-of-stay waste quantity were calculated for the hospital inpatient wards and medical service areas (anatomical pathology, laboratories, radiology, nuclear medicine). Linear regression models assessed the epidemiological impact of COVID, and LOESS analysis modeled the relationship between infectious HW generation and the percentage of COVID-related inpatient days. Average weight of HW per patient was used to estimate the monetary value of CO2 produced.

**Results:**

Preliminary results show that the inpatient days related to COVID nonlinearly influenced the infectious HW generated by wards. PPE usage increased in every context, and the proportion of COVID-related bed-days ranged from 2% to 12% in low-incidence months to 17% to 31% during acute phases. Pre-COVID CO2 production weighted 487 kg per patient and cost 1705€ per-capita, whereas during the pandemic it amounted to 768 kg per patient and cost 2688€ per capita which resulted in a significant increase of 983€ per patient.

**Conclusions:**

In light of the results, HW disposal is an urgent issue that should be addressed by policy makers when implementing new monitoring systems for hospitals. A more adequate disposal of HW could substantially contribute in reducing air pollution and concurrently reduce the economic impact health systems due to the coronavirus pandemic.

**Key messages:**

- The COVID-19 pandemic has brought an increase in hospital waste generation.
- The improvement of HW disposal monitoring system should be considered to avoid reaching critical levels in the near future.

